# How accurate are yield estimates from crop cuts? Evidence from smallholder maize farms in Ethiopia

**DOI:** 10.1016/j.foodpol.2021.102122

**Published:** 2021-07

**Authors:** Frederic Kosmowski, Jordan Chamberlin, Hailemariam Ayalew, Tesfaye Sida, Kibrom Abay, Peter Craufurd

**Affiliations:** a CGIAR Standing Panel on Impact Assessment (SPIA), Addis Ababa, Ethiopia; b International Maize and Wheat Improvement Center (CIMMYT), Kenya; c Trinity College Dublin (TCD), Ireland; d International Food Policy Research Institute (IFPRI), Egypt; e International Maize and Wheat Improvement Center (CIMMYT), Kathmandu, Nepal; f International Maize and Wheat Improvement Center (CIMMYT), Addis Ababa, Ethiopia

**Keywords:** Agricultural systems, Crop production, Crop yields, Measurement errors, Farm survey data, Sampling methods

## Abstract

Agricultural statistics and applied analyses have benefitted from moving from farmer estimates of yield to crop cut based estimates, now regarded as a gold standard. However, in practice, crop cuts and other sample-based protocols vary widely in the details of their implementations and little empirical work has documented how alternative yield estimation methods perform. Here, we undertake a well-measured experiment of multiple yield estimation methods on 237 smallholder maize plots in Amhara region, Ethiopia. We compare yield from a full plot harvest with farmer assessments and with estimates from a variety of field sampling protocols: W-walk, transect, random quadrant, random octant, center quadrant, and 3 diagonal quadrants. We find that protocol choices are important: alternative protocols vary considerably in their accuracy relative to the whole plot, with absolute mean errors ranging from 23 (farmer estimates) to 10.6 (random octant). Furthermore, while most methods approximate the sample mean reasonably well, the divergence of individual measures from true plot-level values can be considerable. We find that randomly positioned quadrants outperform systematic sampling schemes: the random octant had the best accuracy and was the most cost-effective. The nature of bias is non-classical: bias is correlated with plot size as well as with plot management characteristics. In summary, our results advocate that even “gold standard” crop cut measures should be interpreted cautiously, and more empirical work should be carried out to validate and extend our conclusions.

## Highlights

• Accurate measures of yield are essential for evaluating agricultural productivity.
• Substantial heterogeneity in yield estimation protocols has received little critical attention.
• Alternative protocols differ widely in yield estimation accuracy.
• Crop cut from a single, randomly selected octant outperforms other protocols in our study.
• All yield estimation methods in our study suffer from non-classical measurement error.

## 1 Introduction

Agricultural policies rely critically on accurate data for design, monitoring and impact assessments. This is perhaps especially the case in smallholder production systems, where productivity gaps are large and filling them is expected to have large developmental and welfare payoffs. Crop yield estimates constitute a particularly important productivity metric, both at an aggregate level (i.e., in agricultural statistics) as well as in plot-level productivity analysis and impact evaluations of new technologies and policy interventions. Increasing attention to measurement issues in recent years has highlighted shortcomings of farmer reported estimates and generally vindicated crop cut based estimates as important improvements over such subjective and error-prone measures (Carletto and Gourlay, 2019). Yet, in practice, there exists a wide variety of methodological protocols for crop cuts and other yield estimation techniques, with poorly understood implications of methodological choice for yield estimation accuracy. Indeed, the analytical implications of alternative field-based methods of yield measurement have remained relatively unexplored since Casley and Kumar (1988) and Verma et al. (1988).

This paper describes an empirical evaluation of several alternative field-based yield estimation methods indicative of the range of methods currently employed in survey data collection, described in detail below. We seek to answer the following six questions: *i)* How well do alternative methods approximate the sample mean of true yield values? *ii)* How well do alternative methods correlate with true yield values, and with each other? *iii*) What is the magnitude of error for alternative methods? *iv)* By which mechanisms does sampling error arise? *v)* Do methods suffer from non-classical measurement error? and *vi)* How cost-effective are alternative methods?

Our focus on these questions contributes to a growing literature on measurement issues, including land area measurement (Carletto et al., 2015a, Carletto et al., 2015b, Carletto et al., 2013, Dillon et al., 2019, Gourlay et al., 2019, Abay et al., 2019), soil fertility (Berazneva et al., 2018, Kosmowski et al., 2020), crop varietal identification (Kretzschmar et al., 2018, Wossen et al., 2019, Wineman et al., 2020) and animal productivity (Zezza et al., 2016). Despite some progress and improvements in crop production, there remains much work in terms of standardizing and benchmarking crop production and yield measurement. Understanding the nature and breadth of inaccuracies in alternative methods is crucial for computing aggregated estimates of crop productivity; plot-level heterogeneity analysis; and inference making.

Different analytical objectives call for different levels of precision and representativeness. First, obtaining aggregated estimates of crop productivity is typically the mandate of national statistics institutes. With such objectives in mind, the accuracy of the averaged crop yield estimates metrics is given primary importance. In accordance with statistical theory, as the sample size increases the observed values start regressing to the mean and converge to the true mean. It is generally agreed that the mean of the sample reflects the true situation in the population, assuming that errors are random and independent. Randomized control trials that make use of average and standard errors for inference also fall into this category of use. In these settings, a biased sample can still produce a reasonable estimate of yields, on average, if sufficiently powered. There is a second, more stringent data requirement that arises when the analyst’s objective is to perform plot-level productivity analysis (Mishra et al., 2016, Michler et al., 2018, Burke et al., 2019, Amadu et al., 2020). In that case, accurate distribution of yields is needed and each plot must be accurately estimated along with the distribution. Yield estimates need not only be represented at the sample level but also the plot level. Turning again to randomized control trials, it is common to further analyze the effects of a treatment across particular plot characteristics. Subgroup analysis often implies reduced power to detect a similar treatment effect, a situation that might lead to erroneous conclusions. Additionally, while data analysts mostly investigate general and representative plots, recommendation decisions are usually built on individual plot characteristics (Tesfaye et al., 2014, Rurinda et al., 2020) and thus require accurate yield estimates for all fields under evaluation. The implications of measurement errors in crop production for inference making are far-reaching, contributing to a higher chance of spurious relationships and possibly contradicting evidence on scientific debates (Desiere and Jolliffe, 2018, Abay et al., 2019, Gourlay et al., 2019, Lobell et al., 2019).

Our study makes three key contributions to the empirical literature on agricultural productivity measurement. First, it documents the true amount of measurement error and subsequent cost-effectiveness associated with common survey design options for maize yield measurements in small-scale farming systems. Second, it investigates the factors affecting the performance of each sampling method, finding that errors mostly originate from parameters related to the size of n and find little evidence for a substantial effect of yield heterogeneity on sampling errors. Our third contribution is to demonstrate that the direction of bias (i.e. the tendency to over or under-predict true yields) is non-classical: bias is correlated with plot size as well as with plot management characteristics which are typically included in production functions. These non-classical measurement errors do not affect the fit and coefficients of the models when random measurement methods are used.

The rest of the paper is organized as follows. The next section summarizes alternative approaches to yield estimation, along with comments on their relative merits. We then describe our data and empirical approach, after which we present our results for each of the six research questions enumerated above. We conclude with a summary of our main results and their implications for policy and future research.

## 2 Field-based yield estimation methods

A range of methods has been applied to estimate crop production in smallholder farmer’s contexts. The available resources, their context of implementation (on-farm stations *vs* agricultural survey) and the level of precision needed usually dictate the choice of a method. In what follows, we describe five common methods of yield estimates that are commonly applied in the literature.

Farmer estimates: Self-reported measures of yields are commonly collected pre-harvest (farmer predictions) or post-harvest (farmer recall), with most statistical systems in sub-Saharan Africa relying on the latter. Inherently subjective, and conditional on farmers’ experience and education, this method is also highly sensitive to recall bias (Abay et al., 2019, Desiere and Jolliffe, 2018, Gourlay et al., 2019, Casley and Kumar, 1988). Studies have shown substantial disparity between farmers’ estimates and crop cut yields, with some studies reporting patterns of underestimation and overestimation across the distribution of plot sizes (Desiere and Jolliffe, 2018, Gourlay et al., 2019, Wahab, 2019).

Point transect methods: Commonly used in ecological studies and soil sciences, the point transect methods make use of a transect line to collect samples at random or systematic intervals. The technique is helpful for sampling an area relatively quickly with reasonable levels of accuracy (Buckland et al., 2007). A unit of one square meter is usually chosen to collect yield samples.

Crop cut methods: Originating in India in the 1950s (Fermont and Benson, 2011), crop cuts have since become the most widely recommended method for yield estimation (FAO, 1982). Widely used in on-station and on-farm trials, the methods have also been implemented by the Central Statistical Agency (CSA) of Ethiopia since the early 1990s, forming the backbone of its agricultural data. The method consists of laying out a quadrant within the plot and harvesting every crop that falls within the quadrant. The quadrant can be systematically placed, for instance on the center of the plot (Wahab, 2019), or randomly determined. The size of the quadrants is also variable: Sapkota et al. (2016) recommend quadrants of at least 1 m^2^ while Fielding and Riley (1997) used larger quadrants (50–75 m^2^). In a methods experiment in five African countries, Verma et al. (1988) found that two 25 m^2^ subplots overestimated yields in the order of 25–38 percent. A similar upward bias is reported by Diskin (1999) in the context of Bangladesh. Lobell et al. (2020) report that in Uganda, the correlation of estimates from a 64 sq. meter quadrant with the measured yield from a complete plot harvest was only 0.51.

Harvest units: Larger than quadrant, these units usually take the form of rectangles or triangles that span larger areas and are believed to ensure higher representativeness.

Full harvest: Harvesting the full plot provides the most accurate production measure. Full plots harvests are sometimes implemented in research stations or experimental settings (Lobell et al., 2020), although their labor intensiveness and cost render this approach impracticable in most survey settings.

## 3 Data and empirical approach

Data were collected in September 2019 in Dera, Funeteselam and Merawi *woredas* (districts) in Amhara region. These districts are part of the Ethiopian maize belt, an area characterized by higher use of agricultural inputs compared to the rest of the country. In each *woreda*, the survey team coordinated with the extension services to identify farmers that would be willing to participate in the experiment, with the criteria of having a maize plot field ready to be harvested. Listed fields were then surveyed by a team composed of one enumerator and 12–20 laborers hired for the occasion, to implement the plot harvesting. A total of 237 plots validated with two independent area measurements were used for analysis (see Fig. S1).

After collecting farmer consent, information on farmer characteristics, plot management and crop production predictions were collected. Farmers were asked about the quantity they expect to harvest from the selected maize plot. Enumerators were then conducted to the plot to perform six sampling protocols that rely on different sampling units, methods and sizes. The W-walk and transect methods use maize cobs as sampling units; the random quadrant, center quadrant and 3 diagonally-oriented quadrants all use fixed-size areas as units, with sizes ranging from 16 to 48 square meters. The random octant is proportional to the plot size. While all methods are feasible under survey conditions, only the random quadrant and octant rely on probabilistic sampling, with every plant having an equal chance of being selected in the sample.

**Fig. 1:**
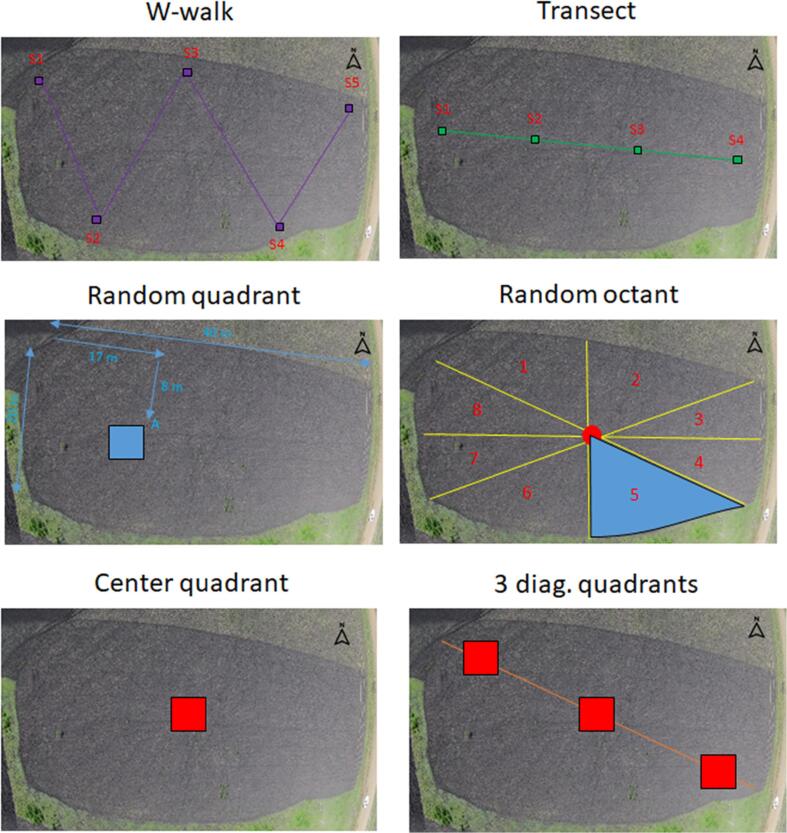
Overview of the yield measurement methods. Note: These methods were preceded by farmer prediction on crop production and followed by a full plot harvest. In the random quadrant and octant methods, the location of the sample unit is only presented as an example.

The six sampling methods described in Fig. 1 were applied in the following order. *Random quadrant:* a randomly placed four by four meters quadrant; *W-walk:* a W path along the two long sides of the plot with three random cobs collected within one square meter at each sampling point. An area of five square meters, corresponding to the five sampling points is used to infer yields at the plot level; *Transect:* a transect path starting at a half distance of the short side, following the long side, and consisting of four samples of three cobs each. Similar to the W-walk, yields are standardized using four square meters; *Center quadrant:* a four-by-four meters quadrant placed at the center of the plot; *3 diagonally-oriented quadrants:* three four-by-four meter quadrants following a diagonal path within the plot. For all methods, maize cobs were weighted and shielded for moisture measurements. All samples remained in place to account for the overlap of methods as well as for the full-harvest final measurement.

These yield measurement methods rely on different sampling units (point, quadrant or harvest unit), sampling methods (systematic *vs* random) and sample sizes. First, the sampling units employed in our survey (point estimate, quadrant or harvest units) introduce variations in unit sizes. Mean share of plot size (or sampling rate) were 0.39% (W-walk), 0.32% (transect), 1.28% (random quadrant and center quadrant), 12.5% (random octant) and 3.89% (3 diagonal quadrants). Adequate size of units is a primary design parameter: sample size must increase with the variability of the true population to achieve any given level of confidence. Second, selecting n sample units at random from N available within the plot is the favored choice when the population is unknown. On the contrary, selecting units systematically consists of ensuring that sample units are well distributed throughout the plot. It is admitted that in contexts where crop yield is homogeneous, systematic sampling could represent an efficient method, providing unbiased and high precision estimates. The dilemma between random *vs* systematic methods should not only be considered in light of crop production heterogeneity, but also in terms of survey duration and costs. Third, our survey design also introduces variations in the number of units (one *vs* three units, for instance in center quadrant *vs* 3 diagonal quadrants) that can be exploited to further understand its effect on accuracy. Ultimately, one should seek the optimal combination of sampling unit, method and size that maximize the accuracy of yield estimates at the plot level.

To assess the accuracy of each method, it is crucial to rely on a separate objective measure of the true distribution of plants, cobs and cob weights within the surveyed plots. To achieve this, the plot was divided into eight harvest units (“octants”) based on regularly spaced bisecting lines, i.e., the plot was divided in a way analogous to cutting a pie into eight similarly sized wedges. Each harvest unit was then measured with measuring tape. Enumerators were in charge of supervising and recording the cutting, husking, counting and weighting of maize cobs within each harvest unit, with the help of 12–20 laborers, depending on plot size. After survey completion, we took advantage of the full harvest data to randomly select one harvest unit, which is labeled as the *Random octant*. Methods duration was carefully recorded for cost-effectiveness analysis (Table 1).

**Table 1:** Yield estimation methods used in this study.

Measure	Description	Average duration (in minutes)
Farmer est.	Farmer estimate	1
W-walk	W-walk with cob collection	38
Transect	Transect with cob collection	35*
Random quadrant	Randomly placed 16 m^2^ quadrant	28
Random octant	Random octant	27**
Center quadrant	16 m^2^ quadrant in plot center	14
3 diag. quadrants	3 × 16 m^2^ quadrants along plot diagonal	41
Full harvest	Full harvest	218

Note: * The transect method also involved picture-taking of maize cobs, with QR codes on a dark background: mean duration is consequently higher than it would be under a strict application of the protocol; ** Enumerator time only, for data recording. The harvest was performed by hired laborers.

For all sampling methods, cobs were shelled, grain moisture was tested and the final yield was adjusted to 12.5% moisture content using the formula Dry Weight = Fresh Weight * (100 – Moisture content)/87.5 and standardized into quintals per hectare.^1^ To account for various harvest unit areas, we used Heron's formula to derive the area of each triangle in terms of the lengths of its sides. The full-harvest plot benchmark was computed by aggregating dry weights from each harvest unit, and subsequently standardized per hectare. Two quality controls were performed on the full harvest benchmark data. Plot-level areas from harvest unit aggregation were compared to total station GPS measurement, convincingly showing an R^2^ of 0.99 (Fig. S1). In Table S1 we also report that 94% of the variation in yield is explained by plant count and mean cob weight.

Several types of analysis are presented in this paper. Following descriptive statistics on the mean and standard deviation of each measurement method (Section 4.1), we assess the strength of correlations between methods using Spearman’s r (Section 4.2). Accuracy metrics are presented in Section 4.3. We then turn our attention to the error-generating mechanisms (Section 4.4) and test the hypothesis of whether measurement errors associated with these different protocols are systematic or random (Section 4.5). We use OLS models to estimate the measurement error per method, calculated as the difference between the standardized full harvest and the standardized method output, in qt/ha. To assess inferential consequences, we estimate a series of log-log (Cobb-Douglas) production functions for each measurement method to evaluate changes in coefficients and fit, in comparison with the full harvest benchmark. In Section 4.6, cost-effectiveness is computed by dividing the average increase in accuracy by the total cost of implementation (Table S2) for a given method. Farmer estimates are used as baseline values for computing the gains in accuracy. We report the results in terms of “additional accuracy per US$1,000 spent.

## 4 Results

### 4.1 How well did the methods approximate sample mean?

**Fig. 2:**
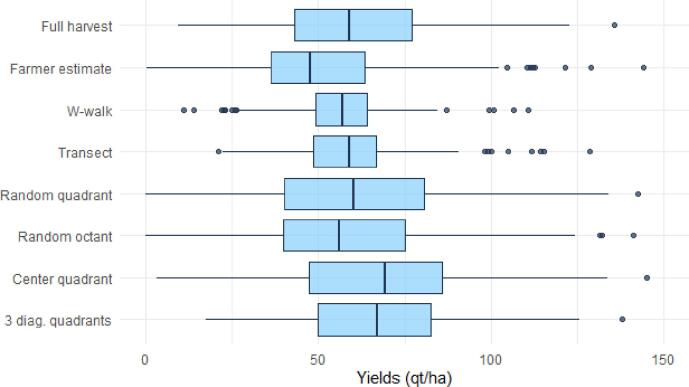
Box plots of mean yields by estimation method. Qt/ha denotes quintals per hectare (1 quintal = 100 kg).

In Fig. 2 we present the box plots of yields for each measurement method. The full harvest plot delivered mean yield estimates of 59.5 qt/ha in the surveyed area, with a standard deviation of 23.9. Farmers’ predictions appear relatively close to the benchmark (Mean = 51.7, S.D. = 24). The entire distribution is slightly lower and several outliers can be observed at the higher side of the distribution.

Cob-based methods (W-walk and transect) both exhibit a similar pattern, failing to capture low-yielding and high-yielding plots in their estimates. Despite a narrower distribution, mean estimates are however in line with the full harvest (56.8 and 59.3 qt/ha respectively). Methods relying on the random allocation of a quadrant or a harvest unit (random octant) deliver mean estimates that do not differ significantly from the full harvest benchmark, at 59.3 and 58.5 qt/ha, respectively. The center quadrant and the 3 diagonal quadrants overestimate the full harvest means, delivering averages>68 qt/ha.

### 4.2 How well did the methods correlate with the benchmark and among each other?

In Table 2, we compute Spearman’s correlations between all measurement methods. It is noteworthy that the highest correlation with the full crop cut benchmark is achieved by the random octant (0.81), followed by the random quadrant (0.63). Both methods moderately correlate (0.55). Methods relying on random allocation of the sampling unit thus appear to deliver the closest correlation with the true value.

**Table 2:** Pairwise correlations between sampling methods.

	Full harvest	Farmer estimate	W-walk	Transect	Random quadrant	Random octant	Center quadrant	3 diag. quadrants
Full harvest	1							
Farmer estimate	0.287***	1						
W-walk	0.256***	0.123	1					
Transect	0.267***	0.089	0.710	1				
Random quadrant	0.629***	0.120	0.258	0.262***	1			
Random octant	0.812***	0.198**	0.189**	0.242***	0.553***	1		
Center quadrant	0.545***	0.129*	0.323	0.393*	0.547***	0.513***	1	
3 diag. quadrants	0.579***	0.125	0.251	0.357***	0.548***	0.551***	0.872***	1

Note: Spearman’s correlation coefficients are calculated on yield measurements from different methods. *** p < 0.001; ** p < 0.01; * p < 0.05.

Systematic, non-random quadrants (center quadrant and 3 diagonal quadrants) have correlation coefficients of 0.54 and 0.57 respectively. Cob-based methods (W-walk and transect), respectively taken at the periphery and the plot center did not perform well, with 0.26 and 0.27 coefficients respectively. It is also apparent that both methods correlate well with each other (0.71). Farmer’s prediction exhibits the weakest correlation with the benchmark (0.26).

Overall, these results suggest the superiority of random methods as well as the importance of sample unit sizes (or crop cut areas) for achieving a higher correlation with the benchmark, as shown by the comparisons between the random quadrants and random octants.

### 4.3 What is the magnitude of errors?

The mean absolute error (MAE) is used to analyze the accuracy of methods. It indicates the average absolute difference between the full harvest benchmark and the alternative method. MAE values relative to the full harvest benchmark ranged from 23 (farmer estimates) to 10.6 (random octant). Farmer estimates, W-walk and transect methods all exhibit low accuracy. The 3 diagonal quadrants method provides the expected improvement in accuracy over a center quadrant (16.1, compared to 19.4 for the center quadrant). The random quadrant, relying on a much smaller area harvested, performed reasonably well, also having desirable properties for a yield measurement method.

Looking at the effect induced by changes in the sampling unit, it is noticeable that as the size of sampling units increase, so does accuracy. However, modifying the number of units (center quadrant *vs* 3 diagonal quadrants) only brings small gains in accuracy, clearly not matching the tripled size of the 3 diagonal quadrants sampling frame. Overall, large divergences exist in the reliability of methods in insuring plot-level representativeness.

### 4.4 By which mechanisms do sampling errors arise?

We now turn our attention to the mechanisms through which sampling errors arise. Understanding these mechanisms will help to improve the design of sampling frames in a variety of agricultural contexts. In particular, whether sampling errors arise because of inadequate sample size and/or plot-level yield heterogeneity are questions of primary importance. If measurement errors correlate with within-plot yield heterogeneity, sampling units should be enlarged and further scattered across plots. Systematic methods might also be encouraged. By contrast, if measurement errors correlate with plot size, larger sampling units should be recommended.

Our dependent variable is the measurement error per method, calculated as the difference between the standardized full harvest and the standardized method output*.* We expect sampling errors to occur for different, potentially overlapping reasons. Sampling errors are likely associated with variables that capture the true distribution of yields. Plot size increases the N units available for sampling and thus introduces errors if the sample size is inadequate. Plot-level heterogeneity in yields can arise due to the heterogeneous number of cobs and variance in cob weights. For both independent variables, we used the coefficient of variation (CV) derived from the eight units composing the full harvest.

Table 3 shows coefficient estimates from linear regression, on samples divided by the direction of measurement error. Three findings regarding the error-generating mechanisms are noteworthy. First, clear divergences exist between the determinants of downward and upward bias. Patterns of overestimation better correlate with variables related to within-plot cob distribution, as reflected by the better fit of the models. Independent variables accounted for 11% to 46% of the variation for the random quadrant and the transect methods respectively. This is a stark contrast with downward bias, for which most models do not explain more than 12% of the variance. This result could suggest that upward bias is more often systematically associated with (unobserved) plot-level variables. Downward bias, by contrast, is more often random and its mechanisms are thus not as clearly identified. Second, the true number of cobs (N) is highly associated with upward errors generated by all measurement methods, as well as with downward errors for the W-walk and the center quadrant methods. This implies that higher-yielding plots tend to generate more upward bias. Similarly interesting is the effect of plot size on measurement errors. An increase in plot size is significantly associated with a decrease in upward bias for all methods, although to a lesser extent for the random methods. Third, contrary to our expectations, the variance of the true population only affects measurement errors to a modest extent. The variance in cobs between the eight harvest units composing the full harvest benchmark is merely associated with an increase in downward errors in the W-walk method. The variance in mean cob weight negatively affects underestimates for the transect, random quadrant, and 3 diagonal quadrants methods (although these associations are only significant at the 0.01 level). To conclude, it is apparent that sampling errors arise primarily from parameters related to the size of N, approximated by plot size and total cob distribution. There is little evidence for an effect of yield heterogeneity on sampling errors.

**Table 3:** Effect of plot cob distribution and variance on downward and upward measurement errors.

	W-walk		Transect		Random quadrant		Random octant		Center quadrant		3 diag. quadrants	
	Errors < 0 (Underest.)	Errors > 0 (Overest.)	Errors < 0 (Underest.)	Errors > 0 (Overest.)	Errors < 0 (Underest.)	Errors > 0 (Overest.)	Errors < 0 (Underest.)	Errors > 0 (Overest.)	Errors < 0 (Underest.)	Errors > 0 (Overest.)	Errors < 0 (Underest.)	Errors > 0 (Overest.)
Total cob count	0.27***	0.71***	0.17	0.76***	−0.04	0.23***	−0.18**	0.22***	−0.01	0.27***	0.06	0.27**
	(0.08)	(0.08)	(0.09)	(0.07)	(0.06)	(0.06)	(0.06)	(0.06)	(0.08)	(0.05)	(0.06)	(0.08)
Area in ha	−66.82*	−360.36***	−27.93	−388.81***	−0.67	−139.66*	92.89**	−89.97*	60.19	−215.46***	44.03	−204.72**
	(30.11)	(53.60)	(31.24)	(48.90)	(35.22)	(52.70)	(29.93)	(40.94)	(49.98)	(57.15)	(43.80)	(62.06)
Cob count (CV)	0.26*	−0.06	−0.02	0.27	0.13	0.13	−0.17	0.19	0.28	0.46*	0.27	0.46
	(0.12)	(0.14)	(0.13)	(0.23)	(0.12)	(0.19)	(0.12)	(0.14)	(0.23)	(0.22)	(0.22)	(0.28)
Mean cob weight (CV)	−0.26	−0.02	−0.21*	−0.08	−0.28*	0.18	−0.08	0.16	−0.33*	−0.06	0.03	−0.14
	(0.14)	(0.08)	(0.08)	(0.09)	(0.13)	(0.17)	(0.12)	(0.11)	(0.16)	(0.08)	(0.08)	(0.22)
Constant	−19.71***	30.22***	−17.10***	23.60***	−11.27*	16.99*	−8.44*	5.18	−31.76**	18.51**	–32.78**	18.30*
	(4.39)	(5.64)	(4.13)	(6.10)	(4.50)	(7.97)	(3.94)	(5.67)	(10.99)	(6.01)	(11.34)	(9.09)
Observations	120	116	130	106	121	115	115	121	148	88	174	62
R^2^	0.11	0.44	0.08	0.46	0.05	0.11	0.12	0.19	0.05	0.29	0.03	0.28

Notes: Table shows coefficient estimates from linear regression, on samples divided by the direction of measurement error. The dependent variable is the measurement error per method, calculated as the difference between the standardized full harvest and the standardized method output, in qt/ha*.* All continuous predictors are mean-centered and scaled by 1 standard deviation. CV = coefficient of variation. Standard errors, in parentheses, are heteroscedasticity robust. *** p < 0.001; ** p < 0.01; * p < 0.05. R^2^ is the coefficient of determination.

### 4.5 Do methods suffer from systematic (non-classical) measurement errors?

Of particular concern for inference is non-classical measurement error (NCME). NCME refers to cases in which the measurement error is correlated with the: *i)* the true value or latent variable; *ii)* the true values of other variables in a model of interest; and *iii)* with the errors in measuring those values (Hyslop and Imbens, 2001). The presence of NCME has the potential to bias analysis and thus jeopardize the validity of analytical conclusions (Bound et al., 2001, Abay et al., 2019).

There are reasons to believe yield measurement methods could be subject to non-classical measurement errors. First, as highlighted by Table 3, we expect errors from most measurement methods to increase with the quantity of harvest (true value). The rationale is that higher-yielding plots certainly offer more potential for large errors than smaller-yielding plots. We do expect random methods to be free from such associations, irrespective of the sampling unit size. Second, we also hypothesize that errors correlate with variables that reveal differences in plot management. These variables do exert an influence on the spatial distribution of yields, an important parameter for sampling accuracy. Farmer estimates stand out as the only method relying on self-elicitation and we expect farmers’ predictions to better correlate with their plot management decisions.

In Table 4, we present regressions of measurement errors on the full harvest latent variable. The striking result from Table 4 is that all methods but the random octant are significantly associated with the latent variable. These associations are extremely strong for farmer estimates, the W-walk and transect methods. For the random quadrant, center quadrant and 3 diagonal quadrants, the R-squared is below 0.06. Fig. S2 provides a visualization of these associations.

**Table 4:** Association of measurement errors with the full harvest latent variable.

	Farmer estimate	W-walk	Transect	Random quadrant	Random octant	Center quadrant	3 diag. quadrants
Full harvest (qt/ha)	0.71***	0.83***	0.82***	0.28 ***	0.10	0.28***	0.26***
	(0.07)	(0.05)	(0.04)	(0.06)	(0.06)	(0.08)	(0.07)
Constant	−34.51***	−46.92***	−48.48***	−16.68***	−4.64	−26.42 ***	−24.58***
	(4.19)	(3.01)	(2.82)	(3.66)	(3.17)	(4.33)	(3.44)
Observations	237	237	237	237	237	237	237
R^2^	0.35	0.64	0.61	0.09	0.02	0.06	0.06

Note: Table shows coefficient estimates from linear regression. The dependent variable is the measurement error per method, calculated as the difference between the standardized full harvest and the standardized method output, in qt/ha. All continuous predictors are mean-centered and scaled by 1 standard deviation. Standard errors, in parentheses, are heteroscedasticity robust. *** p < 0.001; ** p < 0.01; * p < 0.05. R^2^ is the coefficient of determination.

Differences in plot management, particularly through the use of yield-increasing technologies, are likely to have two types of relations with yield measurement errors. First, these could be associated with the true value, hence confounding results presented in Table 4. Second, factors affecting plant growth and related to farm management are frequently used in regression settings and could thus be a source of biased estimates. Accordingly, we now include variables commonly used in econometric analysis: seedling rate, improved seed use, standardized quantities of fertilizer (UREA and DAP), days of labor, irrigation use, and use of erosion-control methods.

The main message from Table 5 is that the addition of variables likely to affect measurement error does not affect the association of errors with the latent variable. It is noticeable that sampling methods with the largest unit size (random octant and 3 diagonal quadrants) show an absence or a less significant relationship. Additionally, two variables correlate with errors in some methods. Irrigated plots are significantly and positively associated with errors in the W-walk and transect methods. Erosion-control methods exhibit the opposite pattern, being negatively associated with errors in the W-walk, transect and the random quadrant. With the exception of the true latent variables, errors from the random octant, center quadrant and 3 diagonal quadrants appear relatively uncorrelated with observables, with only little variation explained.

**Table 5:** Association of measurement errors with plot management and plant growth factors.

	Farmer estimate	W-walk	Transect	Random quadrant	Random octant	Center quadrant	3 diag. quadrants
Full harvest (qt/ha)	0.72***	0.76***	0.75***	0.31***	0.12*	0.26 *	0.21 *
	(0.07)	(0.06)	(0.05)	(0.06)	(0.06)	(0.11)	(0.11)
Area in ha	117.25**	26.31	27.65	−81.07**	30.03	−0.90	29.67
	(35.66)	(21.43)	(19.99)	(29.04)	(26.66)	(34.15)	(31.32)
Seed (kg/ha)	−0.13	0.00	−0.06	−0.04	0.03	0.03	0.07
	(0.08)	(0.04)	(0.04)	(0.04)	(0.03)	(0.05)	(0.05)
Improved seed	−14.15*	2.75	2.43	−1.91	−6.99	2.49	−1.71
	(6.14)	(2.95)	(3.19)	(6.29)	(4.09)	(5.12)	(4.41)
N (kg/ha)	0.00	0.01	0.01	0.00	−0.01	0.00	0.00
	(0.01)	(0.00)	(0.01)	(0.00)	0.00)	(0.01)	(0.01)
Labor (days/ha)	−0.02	0.00	0.01	0.00	0.02*	0.01	0.02
	(0.01)	(0.01)	(0.01)	(0.02)	(0.01)	(0.02)	(0.01)
Irrigated plot: 1 = Yes	−0.13	5.69**	6.22**	−3.14	−3.10	2.61	3.48
	(3.20)	(2.01)	(2.37)	(2.85)	(2.20)	(3.45)	(3.09)
Erosion control methods: 1 = Yes	0.18	−5.42**	−6.28**	−8.21**	0.47	−3.33	−3.65
	(2.82)	(1.84)	(1.97)	(2.79)	(2.03)	(3.35)	(2.98)
Constant	−41.99***	−50.48***	−49.19***	1.81	−11.38	−27.70**	−32.69***
	(8.43)	(4.89)	94.95)	(7.63)	(6.15)	(8.84)	(8.07)
Observations	237	237	237	237	237	237	237
R^2^	0.47	0.67	0.65	0.17	0.08	0.07	0.08

Note: The dependent variable is the measurement error per method, calculated as the difference between the standardized full harvest and the standardized method output, in qt/ha (1 qt = 100 kg). All continuous predictors are mean-centered and scaled by 1 standard deviation. Standard errors, in parentheses, are heteroscedasticity robust. *** p < 0.001; ** p < 0.01; * p < 0.05. R^2^ is the coefficient of determination.

Our analysis suggests the non-classical nature of errors concerns several measurement methods. We now seek to understand how much of a concern this should be for the analyst. In Table 6, we report estimation results from a log-log (Cobb-Douglas) production function, using the full harvest benchmark in the first column, followed by outputs from the alternative measures. The dependent variables are log standardized yield (in qt/ha) obtained from each measurement method. Our main interest in this exercise is to determine whether model fit, associated variables and coefficients change with the dependent variables. The full harvest is used as the benchmark against which other methods are compared.

**Table 6:** Maize production function estimates.

	Full harvest	Farmer estimate	W-walk	Transect	Random quadrant	Random octant	Center quadrant	3 diag. quadrants
Area in ha	−0.61	−2.81	−0.34	−0.39	1.84	−1.25	0.16	−0.34
	(0.68)	(1.56)	(0.40)	(0.37)	(0.99)	(0.84)	(0.69)	(0.64)
LN (Seed (kg/ha)	0.13	0.28*	0.05	0.06	0.17	0.08	0.09	0.03
	(0.08)	(0.14)	(0.05)	(0.04)	(0.10)	(0.09)	(0.08)	(0.06)
Improved seed: 1 = Yes	−0.04	−0.36**	0.05	0.04	0.15	−0.20	0.02	−0.04
	(0.07)	(0.12)	(0.07)	(0.06)	(0.30)	(0.12)	(0.11)	(0.09)
LN (N (kg/ha)	0.10	−0.05	−0.03	−0.01	0.05	0.20	0.04	0.04
	(0.06)	(0.06)	(0.03)	(0.03)	(0.07)	(0.11)	(0.08)	(0.07)
LN (Labor (days/ha))	0.00	0.02	0.00	0.01	0.02	−0.02	0.00	0.00
	(0.02)	(0.04)	(0.01)	(0.01)	(0.03)	(0.02)	(0.02)	(0.02)
Irrigated plot: 1 = Yes	0.25***	0.23	−0.03	−0.04	0.32***	0.27**	0.18**	0.16**
	(0.06)	(0.13)	(0.04)	(0.04)	(0.09)	(0.10)	(0.07)	(0.06)
Erosion control methods: 1 = Yes	−0.01	−0.12	0.09*	0.11**	0.26*	−0.04	0.07	0.05
	(0.06)	(0.12)	(0.04)	(0.04)	(0.10)	(0.08)	(0.06)	(0.05)
Constant	2.94***	3.65***	3.95***	3.86***	2.21***	2.84***	3.42***	3.82***
	(0.46)	(0.70)	(0.30)	(0.28)	(0.66)	(0.76)	(0.55)	(0.50)
Observations	237	237	237	237	237	237	237	237
R^2^	0.14	0.07	0.04	0.06	0.11	0.14	0.05	0.05

Note: Dependent variable = LN (Maize yield (in qt/ha)). Standard errors, in parentheses, are heteroscedasticity robust. *** p < 0.001; ** p < 0.01; * p < 0.05. R^2^ is the coefficient of determination.

The results are presented in Table 6. The production function that uses the full harvest data as a dependent variable shows the highest fit (0.14) while also highlighting the positive and significant effects of irrigation. Remarkably, the fit of the random octant model matches the full harvest one. The coefficient of fertilizer use is the expected positive sign in four of the seven methods we estimated. Irrigation, however, only correlates with random and systematic methods. Interestingly, erosion-control methods correlate with three methods (w-walk, transect and random quadrant) while it shows no relationship with a full harvest production function. Overall, the non-classical measurement errors presented earlier do not affect the significance of independent variables when crop-cut quadrants are used. Notwithstanding, random sampling methods exhibit a higher model fit compared to systematic sampling methods.

### 4.6 How cost-effective are alternative methods?

We finally turn our attention to survey costs, an important parameter for decision-making. Through the choice of a measurement method, survey practitioners have to compose with available resources for maximizing accuracy. Here, the cost per method is derived from the protocol duration multiplied by the overall field survey cost per minute. Field sample protocols were implemented by enumerators, with the exception of the random octant and the full harvest, where laborers were hired for harvesting and husking maize cobs. The costs relative to the duration of the harvest were thus also included in the calculation for both of these methods. See Table S2 for survey costs details.

Given the widespread use and limited cost of collecting farmer’s elicitation on yields, estimates delivered by farmers are used as baseline values for computing the gains in accuracy. We report the results in terms of “additional accuracy per US$1,000 spent” using the average 2019 ETB/USD rate. This metric is calculated by dividing the average increase in accuracy by the total cost of implementation for a given method. For instance, a value of 0.5 indicates that, relative to farmer’s predictions, US$1,000 spent on this sampling protocol will bring an average gain in mean absolute error (MAE) of 0.5 points.

**Fig. 3:**
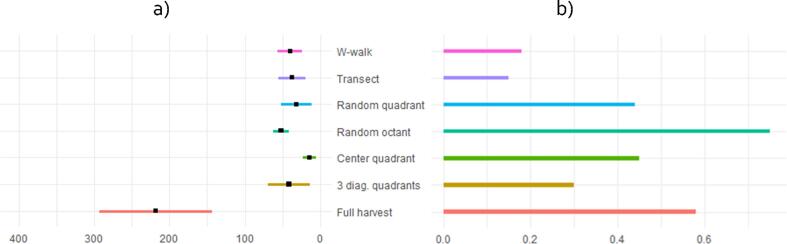
Protocol durations (a) and cost-effectiveness (b) of alternative sampling methods. Mean protocol duration, measured in minutes, is indicated by black dots while the line corresponds to one standard deviation from the mean. Cost-effectiveness is measured by additional changes in accuracy per US$1,000 spent on data collection.

The cost-effectiveness estimates are reported in Fig. 3b, along with the mean and standard deviation of protocol durations (Fig. 3a). Results suggest a clear division of methods into two categories. The first category shows very modest gains in accuracy relative to their cost of implementation. This is true for cob-based methods (W-walk and transect) as well as the 3 diagonal quadrants method. In the second category are found the random quadrant, random octant and the center quadrant: all offer at least 0.4 points gains in mean absolute error per US$1,000 spent in total on data collection. This is interesting as the duration and labor costs involved highly differ between methods. Despite a similar size of 16 m^2^ harvested, randomly allocating the quadrant, as opposed to systematically laying the quadrant in the center resulted in longer protocol duration, as well as higher deviations from the mean. The center quadrant was the quickest method to implement, with a mean duration of 14 min, while the random quadrant took twice as much time on average. Although relatively longer to implement and involving the hiring of daily workers, the random octant comes out as the method delivering the highest accuracy for money.

## 5 Policy implications

Agricultural policies rely on accurate data for design, monitoring and impact. Crop yield measurements are a crucial metric used in aggregated estimates of crop productivity, randomized control trials and plot-level productivity analysis. The results presented in this paper have implications for future research designs, projects monitoring and evaluation, and data analysis.

Our results indicate a clear ranking of the accuracy and cost-effectiveness of the methods we evaluated. While further work should confirm these results, our analysis indicates that methods relying on farmer’s subjective reports as well as transect-based methods can be ruled out as methods of choice. A random 16 m^2^ quadrant outperforms three non-random 16 m^2^ quadrants (i.e., 48 m^2^ total). Higher levels of accuracy can be achieved by increasing the size of crop cuts, as demonstrated by the results obtained with the random octant. Statistical analysis relying on random methods has fewer measurement errors and is free of inferential consequences. These results also demonstrate that even when using the best performing methods, plot-level accuracy should only be assumed, and not guaranteed. This is a concern for analysis that requires accurate data on individual plot characteristics, for instance, recommendation decisions.

## 6 Conclusions

This study has evaluated how well several commonly used yield measurement methods perform relative to one another and a baseline of fully harvested smallholder maize plots in Ethiopia. Several major findings stand out. First, we document large variation in the accuracy of yield estimates across alternative measures, signaling the importance of critically evaluating the performance of different methods. Not all crop cuts are alike in their performance. We find that methods with random sampling locations (such as a randomly located quadrant) outperform methods with a non-random sampling (e.g., central quadrant), even after taking account of other protocol differences. We also find that, as expected, measures with larger sample unit sizes (e.g., crop cut area) outperform methods with smaller sizes. However, these differences are not linear: estimates from three quadrants are not three times better than estimates from a single quadrant. In our analysis, a randomly selected octant (i.e., a “pie slice” segment of the plot covering one-eighth of the approximate total area) outperformed other protocols, particularly after considering the relative cost of implementation.

Secondly, we find that yield estimate errors mostly originate from parameters related to the size of the sample. We find little evidence that intra-field yield heterogeneity affects estimation error. Third, we demonstrate that the tendency to over or under-predict true yields – i.e., the direction of bias – is non-classical: bias is correlated with plot size as well as with plot management characteristics which are typically included in production functions. While such non-classical measurement error raises serious concerns about the validity of existing productivity analysis derived from crop cut data, in practice, the magnitude of these effects may not be large enough to be problematic. In our sample, the presence of non-classical measurement error did not detectably affect estimation results from production functions.

Nonetheless, our finding that higher-yielding plots are associated with more upward bias has important potential implications for understanding farmers’ adoption of agricultural innovations. Given higher productivity often observed on smaller plots in the region (e.g., Assefa et al.’s (2020) analysis of maize farming in Ethiopia), smallholder-dominated systems may have systematic inflations of productivity estimates if such analyses are based on farmer assessments. Such biases may inflate profitability estimates of technologies used on such (for instance, improved seed and fertilizer), which could explain in part the lower-than-expected adoption rates by smallholders. Given the prevalence of very small plots in much of Sub-Saharan Africa (Carletto et al., 2015a), this is a potentially serious distortion of the evidence used to guide policy in the region.^2^ Furthermore, and more generally, to the extent that improved technologies drive yield increases on any size plot, but where the magnitude of such increases are systematically biased upwards, with such bias increasing with yield, then any assessments based on such biased estimates will overstate the productivity and profitability of such technologies – particularly when using mean values of yields which have a right-skewed distribution, as one would expect in systems with relatively limited technology adoption. More empirical research on this question would be valuable.

Although multiple market failures may contribute to agricultural innovations not being adopted (Jack, 2013), the role of low returns on farmer investments remains an important explanatory factor in recent empirical assessments (Burke et al., 2020, Chamberlin et al., 2021). Systematic measurement error could partly explain sub-optimal adoption rates of seemingly profitable modern technologies by smallholders in Africa – i.e., they may not be as profitable as some prior analysis has suggested.^3^ Resolving this question is of central importance to understanding the adoption and impact of agricultural innovations.

More empirical work should certainly be carried out to validate and extend our conclusions. One limitation of our study is the relatively restricted geographical study area. Similar experiments in other farming systems and agroecological conditions will help guide our understanding of the external validity of our results. Another caveat of our study is that we examine only yield estimation in mono-cropped maize fields. Similar work for other crops and mixed-cropping systems would be valuable. Finally, while we endeavored to test as wide a variety of yield estimation protocols as possible, many potential protocol variations remain unevaluated. Our work suggests that additional research attention to alternative randomized crop cut approaches would be particularly valuable.

Our results have potentially important implications for agricultural statistics, for project monitoring and evaluation, for future research design, and the analysis of existing data. Our work indicates that yield estimation protocol decisions matter considerably for analytical outcomes. Identifying the methods that offer the best combination of high accuracy, low cost, and low susceptibility to non-classical sources of bias will be a boon to improving the accuracy of production statistics at scale and the integrity of data collected for monitoring and research purposes. While we are careful not to claim to have definitively identified the optimal yield estimation methods for all settings, the present study does make a valuable initial contribution to a pool of open access data that can eventually provide such definitive statements. With better yield measurements, we can only expect better diagnoses and responses.

## Footnotes

1 A quintal is equivalent to 100 kg.

2 Carletto et al. (2015a) recent study of four African countries found that more than half of all fields are smaller than 0.4 ha, and a quarter of all fields are smaller than 0.2 ha.

3 A separate but related point is the systematic differences in agronomic responses observed in researcher-managed agronomic trials and yields from observational studies of smallholder farms, in which researcher-managed responses typically greatly exceed the responses that most farmers attain (Kravchenko et al., 2017; Laajaj et al., 2020). Such differences also hint that the profitability of farmer investments are lower than what is suggested by most agronomy trials. How the measurement issues we document in this paper map onto these systematic researcher/farmer productivity differences, however, is unclear and would be a useful topic for future research.
