# Supplementary material for: How accurate are yield estimates from crop cuts? Evidence from smallholder maize farms in Ethiopia
Source: Food Policy. 2021 Jul;102:102122. doi: 10.1016/j.foodpol.2021.102122 (PMC8639447; doi:10.1016/j.foodpol.2021.102122)
Supplement: Supplementary data 8 [file mmc8.docx]

# Supporting Information

Dataset S1. Questionnaires

Dataset S2. Datasets

Dataset S3. Analysis replication code

Fig S1. Comparison of measures obtained from two independent plot area measurements. The x axis is the sum of the eight octants, measured with tape by enumerators. Heron's formula was used to derive the area of each triangle in terms of the lengths of its sides. The y axis corresponds to area measured by a mobile team of experts using a total station GPS. n=237 plots


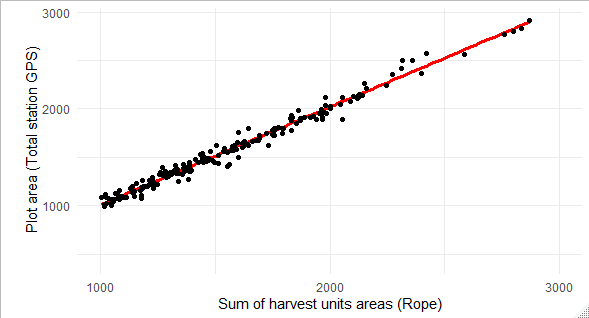


Table S1. Association of plant count and mean cob weight with full harvest maize yields using $\boldsymbol{A}_{\boldsymbol{i}}=\boldsymbol{\alpha}+\boldsymbol{\beta} {\boldsymbol{Cob} \boldsymbol{count}}_{\boldsymbol{i}}+ \boldsymbol{\beta} {\boldsymbol{Mean cob} \boldsymbol{weight}}_{\boldsymbol{i}} {+ \boldsymbol{\varepsilon}}_{\boldsymbol{i}}$

| **Variables** | **Parameter estimates** | **Robust standard error** |
| --- | --- | --- |
| No. of cobs per Ha | 0.001 *** | 0.00 |
| Mean cob weight | 306.8 *** | 8.59 |
| Constant | -41.46 | 1.67 |
| Observations | 237 |  |
| R^2^ | 0.94 |  |

The dependent variable is the standardized maize yields per Ha for plot *i,* measured during the full plot harvest*.* Independent variables are the standardized number of plants per Ha and the mean cob weight for *i*. $\alpha$ is the constant and $\varepsilon$ is the random error term. R^2^ is the coefficient of determination. *** P < 0.0001

Fig S2. Scatterplot of measurement errors (x axis) plotted against the full harvest benchmark (y axis). The red line represents the OLS fit


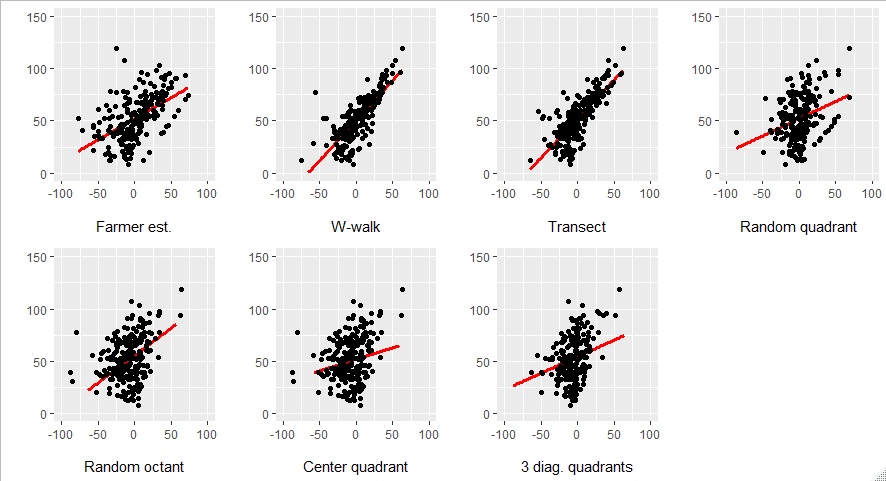


Table S2. Cost of measuring yield at the plot level (2019 USD, using $1 = ETB 31.7)

| A. Survey costs |  |
| --- | --- |
| Cars rentals | $269.5 |
| Gas | $38.5 |
| Interviewer costs | $770 |
| Supervisor costs | $77 |
| Total survey costs | $1,155 |
| Survey cost per minute | $2.4 |
| B. Harvesting costs |  |
| Laborer daily cost | $4.8 |
| Mean number of laborers per plot | 17 |
| Number of plots | 237 |
| Cost per minute | $81.6 |

Table S3. Cost-effectiveness of measurement methods

|  | Average duration (in min) | Total cost (in $) | Change in mean absolute error (MAE) | Reduction in mean absolute error (MAE) per US$1,000 spent |
| --- | --- | --- | --- | --- |
|  |  |  |  |  |
| Farmer estimate | 1 | 570 | / | / |
| W-walk | 38 | 21,670 | 3.92 | 0.18 |
| Transect | 35 | 19,959 | 3.02 | 0.15 |
| Random quadrant | 28 | 15,967 | 6.96 | 0.44 |
| Random octant | 27 | 16,498 | 12.37 | 0.75 |
| Center quadrant | 14 | 7,984 | 3.60 | 0.45 |
| 3 diag. quadrants | 41 | 23,381 | 6.90 | 0.30 |
| Full harvest | 218 | 39,885 | 23.01 | 0.57 |

Note: Farmer estimates are used as baseline values for computing the gains in accuracy. The total cost of random octant and full harvest includes laborer costs. Other protocols were applied by enumerators only.
